# Neonatal group A streptococcal meningitis: a case report and review of the literature

**DOI:** 10.1186/1757-1626-1-108

**Published:** 2008-08-18

**Authors:** Amer A Lardhi

**Affiliations:** 1 Department of Pediatrics, King Fahd Hospital of the University, Alkhobar, Saudi Arabia

## Abstract

**Introduction:**

Group A streptococcus is a rare cause of neonatal meningitis. A review of MEDLINE database since1966 revealed only 15 documented cases of group A streptococcal meningitis in neonates.

**Case report:**

A previously healthy 28 days old male neonate presented with a history of irritability, fever, and focal seizures. Cerebrospinal fluid analysis and culture confirmed the diagnosis of group A streptococcal meningitis. The clinical course was complicated by the development of brain abscess. The patient made full recovery following a surgical drainage of the abscess and a 6-week total course of antibiotics.

**Conclusion:**

Although it is an uncommon organism, clinician should always consider group A streptococcal infection and its potential complications in the differential diagnosis and management of neonatal meningitis.

## Background

Since the introduction of antibiotics, group A streptococcal infection has been an uncommon disease in neonates. Thirty-nine patients with severe neonatal disease caused by group A streptococcus (GAS) have been described since 1966 [1]. Meningitis caused by GAS is reported in only 15 neonates during same period [2-13]. This case report describes a neonate with GAS meningitis seen at a university hospital and provides an overview of well-documented cases in the English-language literature over past 40 years.

## Case report

**Figure 1:**
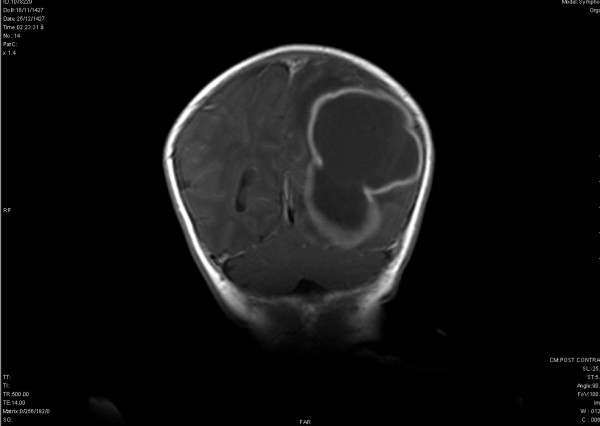
Coronal section of brain MRI with contrast showing a large lobulated collection in the parieto-occipital region.

A 28 days old boy was presented to the Emergency Department with a history of fever, irritability, poor feeding of 1 week duration and right-sided focal seizures on the day of presentation. He was born after an uncomplicated pregnancy and delivery. An elder brother had developed symptomatic pharyngitis with fever, sore and congested throat 10 days prior to the neonate's disease. This pharyngitis, however, was not microbiologically investigated. He was recovered after a one week course of antibiotic. Upon examination, the patient looked ill, and irritable. Vital signs were stable. Anterior fontanel was normal and there was no neurological deficit. The peripheral blood white cells (WBC) were 33,600/mm^3 ^with differential count 61% Neutrophils, 18% Lymphocytes, 6% Monocytes and 10% Eosinophils. C-reactive protein was positive. Cerebrospinal fluid (CSF) was turbid and showed 23,520 WBC/mm^3 ^with 82% Neutrophils, 18% Lymphocytes and 3 red blood cells/mm^3^. CSF protein was 502 mg/dl, Glucose 5 mg/dl (simultaneous blood glucose 98 mg/dl). Latex antigen test was negative for Hemophillus influenzae B, Neisseria meningitides, Escherichia Coli, Streptococcus group B and Streptococcus pneumoniae. Gram positive cocci were seen in the deposit. He was treated with intravenous cefriaxone and vancomycin. The CSF culture yielded group A beta hemolytic Streptococci. Sub-typing of the organism was not available. Vancomycin was discontinued after results of culture. Given the history of focal seizures, head MRI, performed on the seventh hospital day, and showed a large lobulated abscess in the left parieto-occipital region [Figure 1]. The patient underwent drainage of the abscess. No organisms grew in the culture of the pus (presumably owing to the preceding antibiotic treatment). The child was discharged after 6 weeks of treatment. At the age of 13 month, hearing test and neurological examination were normal.

## Discussion

In the pre-antibiotic era, GAS was a major cause of neonatal sepsis and puerperal infections. Meningitis accounted for 10 – 20% of these infections with fatality rate of 95%. Since the advent of antimicrobial therapy, meningitis caused by GAS is rarely reported in adults or children, with less than 1% of all cases of bacterial meningitis [6].

Since the1980's an increase in the incidence of invasive infections caused by GAS has been noted. A review of GAS meningitis in children beyond the neonatal period describes only 31 well-documented patients in world literature in the past 30 years [14].

The present case included a MEDLINE search of the English literature from 1966 revealed only 16 neonates with GAS meningitis [Table 1]. All but two patients in the current review developed late onset (> 7 days of age) neonatal meningitis. Associated conditions included sepsis, erysipelas, necrotizing fasciitis, necrotizing enterocolitis, cellulitis, and respiratory infection. No associated or preceding illness was reported in 6 (40%) patients.

**Table 1:** Group A streptococcal meningitis in neonates.

**Case No.**	**Age/Sex**	**Associated Condition**	**Complication**	**Management**	**Outcome/Sequelae**	**Reference**
1	6 days/M	Erysipelas	None	Penicillin + Kanamycin	Recovered/NA*	2
2	1 Mo./F	None	Seizures	NA	Died	3
3	8 days/M	None	Seizures	NA	Died	3
4	17 days/F	Umbilical sepsis	None	Penicillin	Recovered/NA	4
5	3 days/F	Necrotizing fasciitis, septicemia	None	Penicillin	Recovered	5
6	14 days/F	Erysipelas	Seizures, D.I.C*.	Penicillin	Recovered	6
7	14 days/NA	None	None	Cefotaxime + Ampicillin	Recovered	7
8	26 days/M	Paronychiae, porencephalic cyst	Seizures	Penicillin + Cefuroxime+ Gentamycine	Recovered/Hydrocephalus	8
9	13 days/F	Cellulitis of both feet	Seizures, Hepatitis	Penicillin + Cefuroxime	Died	8
10	1 Mo./F	None	Multiple brain abscess, seizures	Penicillin	Recovered/NA	9
11	1 Mo./F	Sepsis +RSV* Infection	Sepsis, Waterhouse-Friderichsen syndrome	NA	Died	10
12	33 days/F	Pneumonia	NA	Penicillin + Gentamycin	Died	11
13	34 days/NA	None	NA	Penicillin + Gentamycin	Recovered/NA	11
14	1 Mo./NA	NEC*+ Septicemia	NA	Penicillin + Gentamycin	Died	12
15	24 days/F	None	Seizures, brain abscess, cardiorespiratory Insufficiency	NA	Died	13
16	28 days/M	None	Seizures, brain abscess	Ceftriaxone + Vancomycin	Recovered	This case

*D.I.C-Disseminated intravascular coagulation, **NA**-Data not available, **NEC**-Necrotizing entercolitis **RSV**-Respiratory syncetial virus

A 2004 review of GAS invasive infection in the neonates has described 39 patients since 1966 in the world literature [1]. Vertical transmission accounted for the majority of invasive early onset GAS disease in these neonates. Sixty percent of the mothers who delivered infants with early onset of GAS disease developed puerperal sepsis, toxic shock-like syndrome or both in the peripartum period. The mode of transmission in the majority of invasive late onset cases is unknown. Vertical transmission or postnatal acquisition of focal GAS infection such as pharyngitis and episiotomy abscess is a probable source of transmission. There was no a recognizable underlying illness or predisposing factor of GAS meningitis in the present case. However, a family member had developed symptomatic pharyngitis 10 days prior to the onset of the disease, which may have been the source of infection.

GAS is sensitive to a variety of antibiotics administered either alone or in combination. Penicillin was the most commonly prescribed antibiotic for GAS invasive infection in children [14,15]. Burnett et al in his case series reported a favorable outcome when combind clindamycin with beta lactam penicillin [15]. In neonates with GAS meningitis penicillin was used in (77%). Other antibiotics used included amino glycosides, second and third generation cephalosporin, and vancomycin [table 1].

The choice of empiric antibiotic treatment for neonates with meningitis usually involves ampicillin and gentamicin, or ampicillin and cefotaxime.

The clinical course of GAS neonatal meningitis was associated with major complications including seizures in 8 patients (60%) disseminated intravascular coagulopathy (DIC), cardio respiratory insufficiency, hepatitis, and Waterhouse-Frederichsen syndrome. Brain abscess developed in 3 patients [table 1].

Group A streptococcus is an uncommon cause of brain abscess in children and adults. Etiology of brain abscess in the reported cases includes meningitis, contiguous spread from a middle ear infection, facial furuncles and hematogenous spread from distant site.

In neonates brain abscesses are very rare. It is usually occurs as complications of bacterial meningitis or bacteremia. Maternal factors include mastitis and genitourinary tract infection can be an important source of neonatal brain abscesses. They are most often caused by gram negative organisms. The abscesses are often large and may be multiple. Symptoms of seizures, signs of sepsis and bulging fontanels are frequently seen in neonatal brain abscesses.

Eight (50%) patients with neonatal GAS meningitis died. This is higher than the mortality rate reported in neonates with meningitis caused by several other types of pathogens, and the mortality rate of GAS meningitis reported in children beyond the neonatal period [14]. No neurological sequelae were reported in 4 patients on whom follow up data is available.

## Conclusion

GAS meningitis remains an uncommon but serious disease affecting mostly older neonates. Parent and siblings of the patients constitute the source of infection and may unknowingly infect the neonates. Since invasive infection is on the increase, clinicians should always consider GAS in the differential diagnosis of neonatal sepsis and meningitis. Prompt and appropriate treatment may reduce complications and mortality.

## Consent

Written informed consent was obtained from the patient's parent for publication of this case report and accompanying image. A copy of the written consent is available for review by the Editor-in Chief of this journal.

## Competing interests

The author declares that they have no competing interests.
